# Association of social and cognitive impairment and biomarkers in autism spectrum disorders

**DOI:** 10.1186/1742-2094-11-4

**Published:** 2014-01-08

**Authors:** Altaf Alabdali, Laila Al-Ayadhi, Afaf El-Ansary

**Affiliations:** 1 Biochemistry Department, Science College, King Saud University, P.O box 22452, Zip code 11495 Riyadh, Saudi Arabia; 2 Autism Research and Treatment Center, King Saud University, Riyadh, Saudi Arabia; 3 Shaik AL-Amodi Autism Research Chair, King Saud University, Riyadh Postal code 99, Saudi Arabia; 4 Department of Physiology, Faculty of Medicine, King Saud University, Riyadh Postal code 29, Saudi Arabia; 5 Medicinal Chemistry Department, National Research Centre, Tahrir street, Dokki, Guiza postal code 12622, Egypt

**Keywords:** Autism spectrum disorders, Childhood autism rating scale, Interferon-γ-induced protein-16, Neuroinflammation, Neurotransmitters, Oxytocin, Social responsiveness scale

## Abstract

**Objectives:**

The neurological basis for autism is still not fully understood, and the role of the interaction between neuro-inflammation and neurotransmission impairment needs to be clearer. This study aims to test the possible association between impaired levels of gamma aminobutyric acid (GABA), serotonin, dopamine, oxytocin, and interferon-γ-induced protein-16 (IFI16) and the severity of social and cognitive dysfunctions in individuals with autism spectrum disorders.

**Materials and methods:**

GABA, serotonin, dopamine, oxytocin, and IFI16 as biochemical parameters related to neurochemistry and inflammation were determined in the plasma of 52 Saudi autistic male patients, categorized as mild-moderate and severe as indicated by their Childhood Autism Rating Scale (CARS) or social responsiveness scale (SRS), and compared to 30 age- and gender-matched control samples.

**Results:**

The data indicated that Saudi patients with autism have remarkably impaired plasma levels of the measured parameters compared to age and gender-matched controls. While serotonin in platelet-free plasma and dopamine did not correlated with the severity in social and cognitive dysfunction, GABA, oxytocin, and IFI16 were remarkably associated with the severity of both tested scores (SRS and CARS).

**Conclusions:**

The relationship between the selected parameters confirms the role of impaired neurochemistry and neuro-inflammation in the etiology of autism spectrum disorders and the possibility of using GABA, oxytocin, and IFI16 as markers of autism severity. Receiver operating characteristic analysis together with predictiveness diagrams proved that the measured parameters could be used as predictive biomarkers of clinical symptoms and provide significant guidance for future therapeutic strategy to re-establish physiological homeostasis.

## Introduction

Autism spectrum disorders (ASDs) are a behaviorally defined group of disorders characterized by impaired social skills, impaired language, and restricted areas of interest [1]. Additional features may include poor eye contact [2], repetitive behavior [3], sensory modulatory dysfunction [4], and varying levels of cognition and motor disturbances [5,6]. Clinical signs are usually present at the age of 3 years, but prospective studies of infants at risk have demonstrated that deficits in social responsiveness, communication, and play could be present early, at the age of 6–12 months [7]. However, the condition is often missed and not diagnosed until later in a child’s life, especially when the condition is mild or even moderate in severity. There has been increasing interest in developing effective interventions for young children with autism since the evidence suggests that early intervention programs are indeed beneficial for children with autism, often improving developmental functioning and decreasing maladaptive behaviors and symptom severity and can also improve outcomes in later years for many individuals [8].

Abnormalities in neurotransmitter systems have frequently been reported in autism. Clinical observations include altered levels of various neurotransmitters compared to controls, including alterations in dopamine metabolism [9] and dysregulations of serotonergic systems [10-13]. Expression of several types of gamma aminobutyric acid (GABA) receptors is altered in the brains of subjects with autism, with levels being significantly reduced in autism compared to controls [14-16]. In addition, impaired vasopressin/oxytocin metabolism was also reported in patients with autism compared to controls [17]. Moreover, studies have revealed increased levels of pro-inflammatory cytokines in brain, cerebrospinal fluid (CSF), and blood from children with ASDs [18].

Recently, there has been increasing interest in the role of chronic inflammation in neurological disorders. The progressive increase of clinical and experimental research into inflammation has been initiated by knowledge that inflammation-induced changes in brain disorders are not limited to specific neurotransmitter abnormalities but reflect multifunctional changes in the oxidative/antioxidant status, and immune, endocrine, and neurotransmitter circuits in the brain [19]. Neuroinflammation was first identified in a study of post-mortem samples from 11 individuals with autism aged 5–44, in which activated astrocytes and microglial cells together with abnormal inflammatory cytokines were found [20,21]. Additional support for the presence of tissue neuroinflammatory-based changes in brains of people with autism comes from the various studies showing reduction in Purkinje cell numbers, possibly due to inflammation-induced oxidative stress leading to an increased excitation/inhibition ratio that could potentially be due to imbalanced glutamatergic and GABAergic systems [13,14]. Although it is accepted that neuroinflammation can alter neurotransmitter function and its transporters [22] producing cognitive, behavioral, and psychiatric impairments, and that excess central nervous system (CNS) glutamate levels can induce neurotoxicity-mediated inflammation [23-26], the interaction between these two pathologic mechanisms have not been clarified in patients with autism.

A number of studies have shown that inflammatory cytokines, including tumor necrosis factor (TNF)-α, interferon (IFN)-γ, IL6, IL8, and IL12, are elevated in blood mononuclear cells, plasma, serum, CSF, and brain of autistic subjects [27-30]. Among these cytokines, IFN-γ orchestrates the trafficking of specific immune cells to sites of inflammation through up-regulating protein mediators’ expression such as IFN-γ-induced protein-16 (IFI16). Additionally, TNF-α synergistically regulates the expression of these in adhesion and chemokine molecules [31].

This work is an attempt to understand the interaction between GABA, serotonin, dopamine, and oxytocin as important neurotransmitters and IFI16 as an inflammatory mediator that has not been extensively studied in ASD compared to other cytokines. Moreover, a biochemical correlate to the social responsiveness scale (SRS) and the Childhood Autism Rating Scale (CARS) as measures of social and cognitive impairments in patients with autism was screened among the measured parameters.

## Materials and methods

### Subjects

This cross-sectional study was conducted on 52 male children who had ASD, recruited from the Autism Research and Treatment Center, Faculty of Medicine, King Saud University, Riyadh, Saudi Arabia; 40 were non-verbal and 12 were verbal. Their ages ranged between 3 and 12 years (mean SD = 7.0 ± 2.34 years). The control group comprised 30 age and sex-matched apparently healthy children with mean age 7.2 ± 2.14 years. The patients met the diagnostic criteria of ASD according to the 4^th^ edition of the Diagnostic and Statistical Manual of Mental Disorders [1]. The controls were normally developing, healthy children, unrelated to the autistic subjects and without any of the exclusion criteria. These children were the healthy older siblings of healthy infants who were attending the Well Baby Clinic at King Khalid University Hospital for routine check-up of their growth parameters. They had no clinical indications of infectious disease or neuropsychiatric disorders. All participants had normal results for urine analysis and sedimentation rate. The local Ethical Committee of the Faculty of Medicine, King Saud University, Riyadh, Saudi Arabia, approved this study. In addition, an informed written consent of participation in the study was signed by the parents or the legal guardians of the investigated subjects according to the Helsinki principles.

The CARS score was completed as a further measurement of the severity of disease. CARS rates the child on a scale from one to four in each of 15 areas (relating to people; emotional response; imitation; body use; object use; listening response; fear or nervousness; verbal communication; non-verbal communication; activity level; level and reliability of intellectual response; adaptation to change; visual response; taste, smell and touch response; and general impressions). According to the scale, children who have scored 30–36 have mild to moderate autism (n = 23), while those with scores ranging between 37 and 60 points have severe autism (n = 27) [32]. Regarding the SRS, a questionnaire was completed in 15 to 20 minutes. A total score of 76 or higher is considered severe and strongly associated with a clinical diagnosis of autistic disorder. A score of 60–75 is interpreted as falling in the mild to moderate range of social impairment [33]. Participants were excluded from the study if they had a diagnosis of fragile X syndrome, epileptic seizures, obsessive-compulsive disorder, affective disorders, or any additional psychiatric or neurological diagnoses.

### Sample collection

After overnight fast, 10 mL blood samples were collected from both groups in test tubes containing sodium heparin as anticoagulant. Tubes were centrifuged at 3,500 rpm at room temperature for 15 minutes. Plasma and red blood cells were obtained and deep frozen (at −80°C) until analysis time.

### Biochemical analysis

#### Determination of serotonin

The ELISA diagnostic kit used for serotonin was a product of IBL International, Germany. The assay procedure follows the basic principle of competitive ELISA whereby there is competition between a biotinylated and a non-biotinylated antigen for a fixed number of antibody binding sites. The amount of biotinylated antigen bound to the antibody is inversely proportional to the analyte concentration in the sample. When the system is in equilibrium, the free biotinylated antigen is removed by a washing step and the antibody-bound biotinylated antigen is determined by use of anti-biotin alkaline phosphatase as marker and p-nitro phenyl phosphate as a substrate. Quantification of unknowns is achieved by comparing the enzymatic activity of unknowns with a response curve prepared by using known standards.

#### Determination of dopamine

The quantitative determination of dopamine in human plasma was measured using ELISA diagnostic kit, a product of American Laboratory Products Company (ALPCO), United States.

Dopamine was extracted by using a cis-diol-specific affinity gel, acylated and then derivatized enzymatically. The competitive ELISA kit used the microtiter plate format. The antigen is bound to the solid phase of the microtiter plate. The derivatized standards, controls, and samples and the solid phase-bound analytes competed for a fixed number of antiserum binding sites. After the system was in equilibrium, free antigen and free antigen-antiserum complexes were removed by washing. The antibody bound to the solid phase was detected by an anti-rabbit IgG-peroxidase conjugate using tetramethylbenzidine as a substrate. The reaction was monitored at 450 nm. Quantification of unknown samples was achieved by comparing their absorbance with a reference curve prepared with known standard concentrations.

#### Determination of gamma aminobutyric acid (GABA)

The quantitative determination of GABA in human plasma was measured using ELISA diagnostic kit, a product of Immunodiagnostic AG, Germany. This assay was based on the method of competitive enzyme linked immunoassays. The sample preparation included the addition of a derivatization reagent for GABA. Afterwards, the treated samples were incubated in wells of a microtiter plate coated with a polyclonal antibody against GABA-derivative, together with assay reagent containing GABA-derivative (tracer). During the incubation period, the target GABA in the sample competed with the tracer for the binding of the polyclonal antibodies on the wall of the microtiter wells. GABA in the sample displaced the tracer out of the binding to the antibodies. Therefore, the concentration of antibody-bound tracer was inversely proportional to the GABA concentration in the sample.

During the second incubation step, a peroxidase conjugate was added to each microtiter well to detect the tracer. After being washed away, the unbound components tetramethylbenzidine were added as a peroxidase substrate. Finally, the enzymatic reaction was terminated by an acidic stop solution. The color changed from blue to yellow, and the absorbance was measured at 450 nm using a spectrophotometer. The intensity of the yellow color was inversely proportional to the GABA concentration in the sample; therefore, a high GABA concentration in the sample reduced the concentration of antibody-bound tracer and lowered the photometric signal. A dose response curve of absorbance unit (optical density at 450 nm) vs. concentration was generated using the values obtained from the standards. GABA present in the patient samples (EDTA plasma) was determined directly from this curve.

#### Determination of oxytocin

The ELISA assay kit was a product of BioSource, USA. This immunoassay kit allows the *in vitro* quantitative determination of human oxytocin concentrations in plasma. The microtiter plate provided in this kit had been pre-coated with an antibody specific to oxytocin. Standards or samples were then added to the appropriate microtiter plate wells with a horseradish peroxidase (HRP)-conjugated antibody and incubated. Substrate solutions were then added to each well. The enzyme-substrate reaction was terminated by the addition of a sulfuric acid solution and the color change was measured spectrophotometrically at 450 nm. The concentration of oxytocin in the samples was then determined by comparing the optical density of the samples to the standard curve.

#### Determination of interferon-γ-inducible protein 16 (IFI16)

The human IFI16 ELISA kit was provided with by BioSource. This immunoassay kit allows for the *in vitro* quantitative determination of human IFI16 concentrations in plasma. The IFI16 ELISA kit applied the quantitative sandwich enzyme immunoassay technique. The microtiter plate had been pre-coated with a monoclonal antibody specific for IFI16. Standards or samples were then added to the microtiter plate wells and IFI16, if present, bound to the antibody pre-coated wells. In order to quantitatively determine the amount of IFI16 present in the sample, a standardized preparation of HRP-conjugated polyclonal antibody, specific for IFI16, was added to each well to “sandwich” the IFI16 immobilized on the plate. The microtiter plate underwent incubation, and then the wells were thoroughly washed to remove all unbound components. Next, substrate solutions were added to each well. The enzyme (HRP) and substrate were allowed to react over a short incubation period. Only those wells that contained IFI16 and HRP-conjugated antibody exhibited a change in color. The enzyme-substrate reaction was terminated by the addition of a sulfuric acid solution and the color change was measured spectrophotometrically at a wavelength of 450 nm. A standard curve was plotted relating the intensity of the color (optical density) to the concentration of standards. The IFI16 concentration in each sample was interpolated from this standard curve.

### Statistical analysis

A SPSS computer program was used. Results were expressed as mean ± SD and all statistical comparisons were made by means of independent *t*-test with *P* ≤0.05 considered as significant. Receiver Operating Characteristic (ROC) analysis was performed as a comprehensive way to measure the accuracy of the studied markers. The area under the curve (AUC) provides a useful metric to compare different biomarkers. Whereas an AUC value close to 1 indicates an excellent diagnostic and predictive marker, a curve that lies close to the diagonal (AUC = 0.5) has no diagnostic utility. AUC close to 1 is always accompanied by satisfactory values of specificity and sensitivity of the biomarker. Moreover, the predictiveness diagrams of the measured parameters were drawn in which the x axis represents percentile rank of the biomarker, the y axis represents the probability of identifying the disease, and the horizontal line is the prevalence of the disease using a Biostat 16 computer program.

## Results

**Figure 1:**
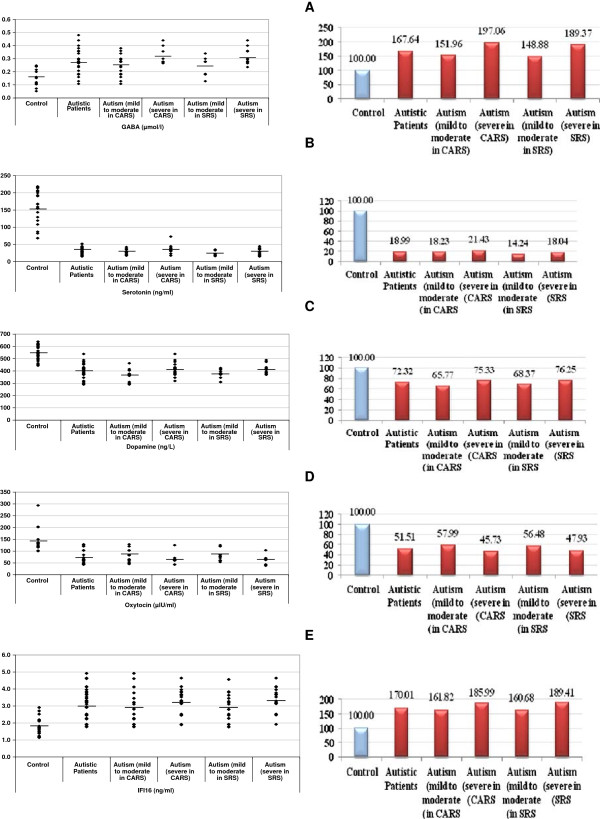
**Left side: (A) GABA (μmol/L), (B) serotonin (ng/mL), (C) dopamine (ng/L), (D) oxytocin (μLU/mL), (E) IFI16 (ng/mL) levels of control and autistic groups.** The mean value for each group is designated by a line. **Right side:** Percentage change of **(A)** GABA, **(B)** serotonin, **(C)** dopamine, **(D)** oxytocin, and **(E)** IFI16 in autistic group compared to control.

Levels of GABA, serotonin, dopamine, oxytocin, and IFI16 were compared between patients with different severity of autism (mild-moderate or severe) and age-matched control subjects. Patients were classified according to their recorded SRS and CARS scores (Table 1). Data are presented as a mean ± SD of a maximum number of 52 patients with ASDs compared to 30 controls, and the significant difference between both groups and subgroups of patients with autism was presented in the table. It was noticed that the five measured parameters differed significantly either between patients and controls or between subgroups of patients showing different levels of cognitive and social impairment (mild-moderate and severe). Figure 1A–E demonstrates individual data distribution around the mean value represented as straight line for the five studied parameters. Moreover, they represent the increase of GABA and IFI16 and decrease of serotonin, dopamine, and oxytocin as percentage of ASD patients compared to controls.

**Table 1:** GABA (μmol/L), serotonin (ng/mL), dopamine (ng/L), oxytocin (μLU/mL), and IFI16 (ng/mL) levels of control and autistic groups

**Parameters**	**Group**	**n**	**Mean ± SD**	***P *** **value**^**a**^	***P *** **value**^**b**^
**GABA (μmol/L)**	Control	30	0.16 ± 0.04		
	Patients with autism	30	0.27 ± 0.09		0.001
	Autism (mild to moderate in CARS)	21	0.24 ± 0.07	0.016	0.001
	Autism (severe in CARS)	9	0.32 ± 0.07		0.001
	Autism (mild to moderate in SRS)	12	0.24 ± 0.07	0.010	0.002
	Autism (severe in SRS)	15	0.31 ± 0.06		0.001
**Serotonin (ng/mL)**	Control	30	154.83 ± 41.75		
	Patients with autism	48	29.40 ± 8.95		0.001
	Autism (mild to moderate in CARS)	22	28.23 ± 6.12	0.043	0.001
	Autism (severe in CARS)	25	33.18 ± 9.58		0.001
	Autism (mild to moderate in SRS)	15	22.04 ± 5.41	0.017	0.001
	Autism (severe in SRS)	21	27.92 ± 7.83		0.001
**Dopamine (ng/L)**	Control	27	548.78 ± 54.12		
	Patients with autism	28	396.86 ± 60.97		0.001
	Autism (mild to moderate in CARS)	11	360.93 ± 54.78	0.019	0.001
	Autism (severe in CARS)	17	413.41 ± 54.05		0.001
	Autism (mild to moderate in SRS)	11	375.19 ± 40.10	0.019	0.001
	Autism (severe in SRS)	12	418.46 ± 41.76		0.001
**Oxytocin (μLU/mL)**	Control	30	139.22 ± 36.62		
	Patients with autism	50	71.71 ± 18.09		0.001
	Autism (mild to moderate in CARS)	27	80.74 ± 18.78	0.001	0.001
	Autism (severe in CARS)	23	63.67 ± 14.72		0.001
	Autism (mild to moderate in SRS)	20	78.63 ± 17.16		0.001
	Autism (severe in SRS)	18	66.72 ± 17.92		0.001
**IFI16 (ng/mL)**	Control	30	1.75 ± 0.42		
	Patients with autism	56	2.97 ± 0.84		0.001
	Autism (mild to moderate in CARS)	29	2.82 ± 0.85	0.048	0.001
	Autism (severe in CARS)	25	3.25 ± 0.64		0.001
	Autism (mild to moderate in SRS)	21	2.80 ± 0.78	0.028	0.001
	Autism (severe in SRS)	21	3.31 ± 0.64		0.001

^a^*P* value between mild to moderate and severe in CARS and SRS.

^b^*P* value between control and autistic groups.

Table 2 demonstrates the ROC analysis data as AUC, cutoff values, specificity, and sensitivity of the measured parameters. All parameters exhibited AUC values close to 1 and satisfactory values of accuracy presented as high specificity and sensitivity.

**Table 2:** ROC curve of GABA (μmol/L), serotonin (ng/mL), dopamine (ng/L), oxytocin (μLU/mL), and IFI16 (ng/mL) in autistic groups

		**Patients with autism**	**CARS**		**SRS**	
**Parameters**			**Mild to moderate**	**Severe**	**Mild to moderate**	**Severe**
**GABA (μmol/L)**	Area under the curve	0.883	0.843	1.000	0.864	0.993
	Best cutoff value	0.170	0.170	0.257	0.170	0.257
	Sensitivity%	90.0%	85.7%	100.0%	91.7%	93.3%
	Specificity%	80.0%	80.0%	100.0%	80.0%	100.0%
**Serotonin (ng/mL)**	Area under the curve	1.000	1.000	0.997	1.000	1.000
	Best cutoff value	59.925	55.225	56.400	51.700	56.400
	Sensitivity%	100.0%	100.0%	96.0%	100.0%	100.0%
	Specificity%	100.0%	100.0%	100.0%	100.0%	100.0%
**Dopamine (ng/L)**	Area under the curve	0.968	0.993	0.959	1.000	0.969
	Best cutoff value	477.771	463.014	490.207	433.682	490.207
	Sensitivity%	92.9%	100.0%	94.1%	100.0%	100.0%
	Specificity%	88.9%	92.6%	85.2%	100.0%	85.2%
**Oxytocin (μLU/mL)**	Area under the curve	0.981	0.973	0.990	0.979	0.989
	Best cutoff value	92.105	92.105	85.555	92.105	109.720
	Sensitivity%	90.0%	85.2%	95.7%	90.0%	100.0%
	Specificity%	100.0%	100.0%	100.0%	100.0%	90.0%
**IFI16 (ng/mL)**	Area under the curve	0.933	0.916	0.973	0.918	0.978
	Best cutoff value	1.750	1.750	2.320	1.860	2.330
	Sensitivity%	100.0%	100.0%	92.0%	90.5%	95.2%
	Specificity%	70.0%	70.0%	90.0%	80.0%	90.0%

**Figure 2:**
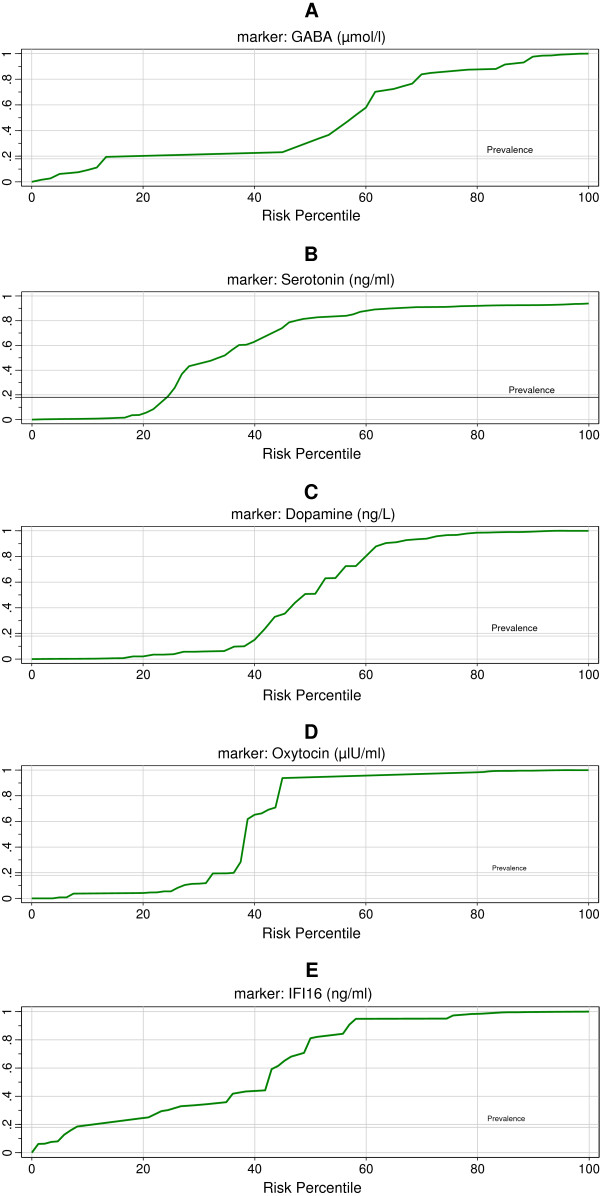
Predictiveness curve of (A) GABA, (B) serotonin, (C) dopamine, (D) oxytocin, and (E) IFI16.

Figure 2A–E demonstrates the predictiveness curves as an assessment of the performance of GABA, serotonin, dopamine, oxytocin, and IFI16 in autism risk prediction in the Saudi population. The four measured parameters showed adequate predictive power.

**Figure 3:**
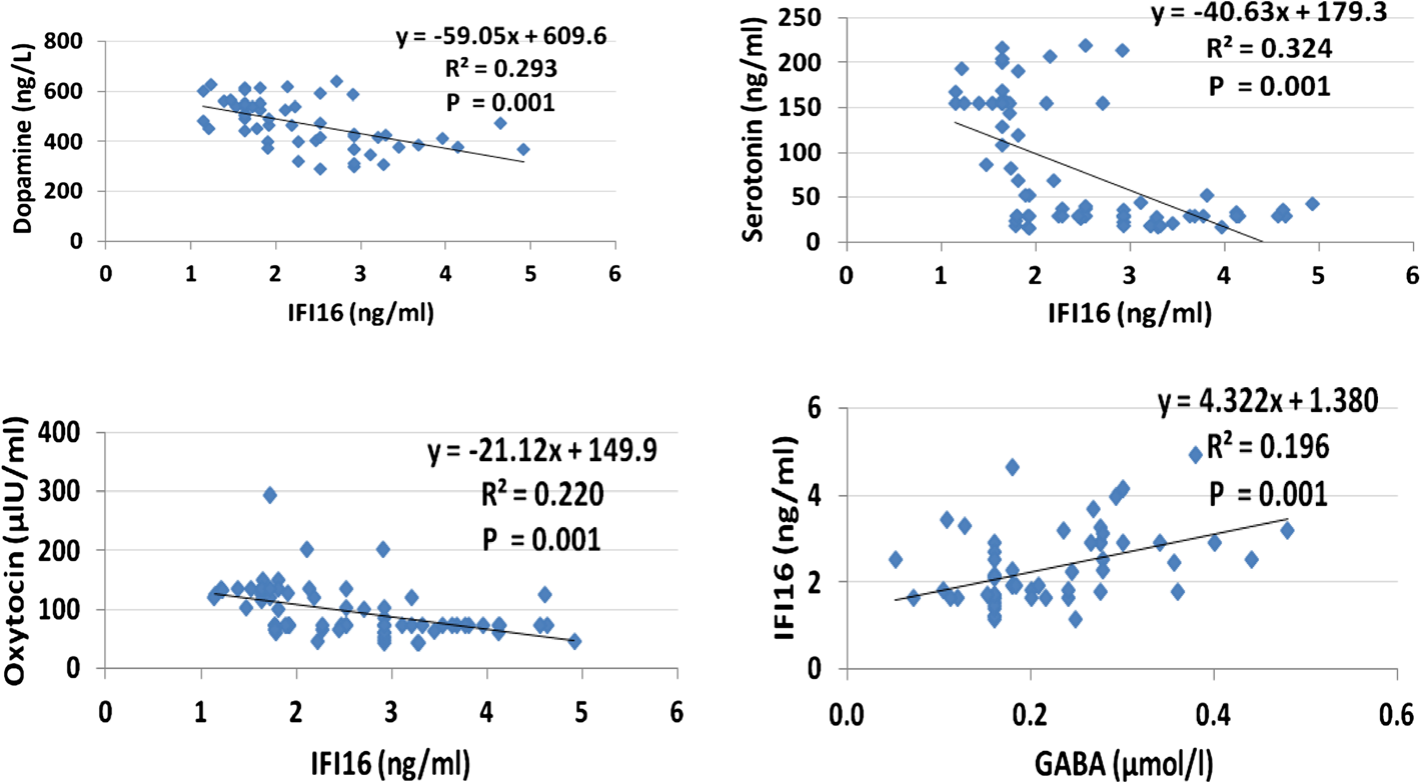
Pearson correlations between IFI16, neurotransmitters, and oxytocin.

Figure 3 demonstrates the correlation between IFI16 as a biomarker of inflammation and GABA, serotonin, dopamine, and oxytocin. It was observed that inflammation positively correlated with GABA and negatively correlated with the other three measured parameters.

**Figure 4:**
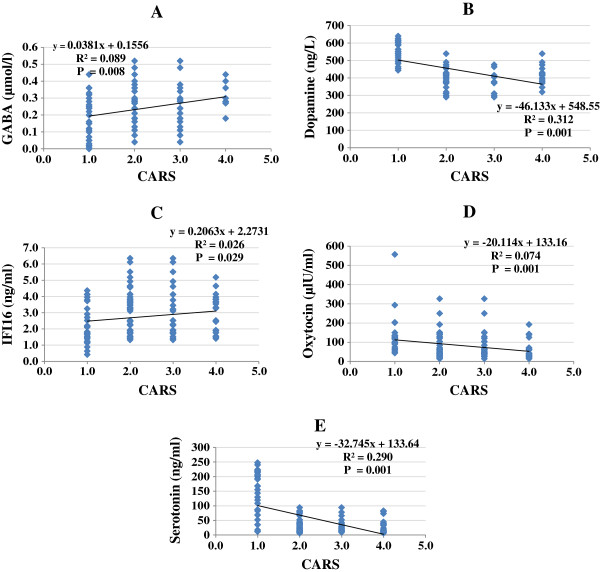
(A-E): Pearson correlations between CARS and the different measured parameters.

**Figure 5:**
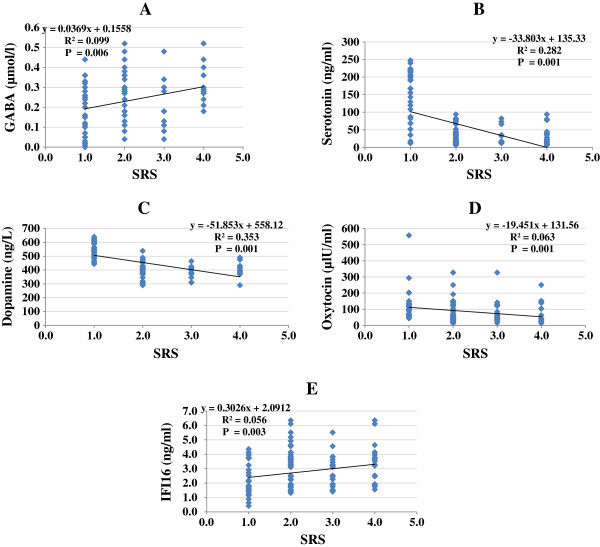
(A-E): Pearson correlations between SRS and the different measured parameters.

Figures 4 and 5 demonstrate the correlation between cognitive and social impairment (CARS and SRS), respectively, and the five measured parameters. Both scores were positively correlated with GABA and IFI16 and negatively correlated with serotonin, dopamine, and oxytocin.

### Correlations

By using a statistical analysis program (SPSS) which includes the Pearson correlation test, the correlation was done between all parameters; the results showed that there was a correlation significance difference between the parameters shown in Table 3.

**Table 3:** Pearson correlation parameters

**Parameters**	**R (Pearson correlation)**	**Sig.**	
GABA (μmol/L) – IFI16 (ng/mL)	0.443	0.001	P^a^
IFI16 (ng/mL) – Dopamine (ng/L)	−0.542	0.001	N^b^
IFI16 (ng/mL) – Serotonin (ng/mL)	−0.570	0.001	N^b^
IFI16 (ng/mL) – Oxytocin (μLU/mL)	−0.470	0.001	N^b^

^a^Positive correlation.

^b^Negative correlation.

## Discussion

Plasma as a complex body fluid containing proteins, peptides, lipids and metabolites reflects the physiological activity and pathology in various organs, including the CNS. In humans, about 500 mL of CSF is absorbed into the blood daily, making blood a suitable source of neurodegenerative or neurodevelopmental disease biomarkers [34].

Although neurotransmitter assessment can be a useful tool in any clinical practice, immune system and nervous system activity must be considered and examined as a single system functioning in parallel. It is well established that neurological and immunological abnormalities exist in autistic individuals; however, the relationship between neural and immune function has not been emphasized enough.

Recently, GABA, as the major inhibitory transmitter in the CNS, has been implicated in the pathophysiology of ASDs [35]. Table 1 and Figure 1A demonstrate the significantly higher level of plasma GABA in patients with autism compared to controls with a possibility of concomitant low brain GABA levels. This suggestion is supported by many previous studies in which GABAergic inhibitory problems, reduced GABAA-receptor binding in hippocampus, neocortex and cerebellum, and protein levels of several GABA receptor subunits have been reported in autism [36]. The association between elevated plasma GABA and severity of autism (CARS and SRS) can be easily seen in Table 1. Severe autistics show higher plasma GABA levels compared to mild-moderate patients. This could be supported by the previous studies which show that male mice that have been socially isolated for more than 4 weeks demonstrate a reduced responsiveness of GABA_A_ receptors to the administrations of GABA-mimetic drugs [37]. Moreover, knockout mice lacking the GABRB3 gene display seizures, hypersensitive behavior, learning and memory deficits, and poor motor skills as features common to the Angelman syndrome, a disorder with some similarities to autism [38]. Additionally, the association between elevated GABA levels and impaired cognition reported in the present study could be supported by a recent study in which treatment with GABA_A_ receptor antagonist pentylenetetrazole elicited long-lasting (>1 week) normalization of cognitive function in young and aged mice [39].

As serotonin has a wide range of effects on normal physiological function including circadian rhythms, appetite, mood, sleep, anxiety, cognition, and memory formation, its impaired level could be easily related to many autistic features such as sleep disturbances and social and cognition impairment [40]. In the present study, platelet-free plasma serotonin levels were significantly lower in patients with autism compared to control subjects (Table 1 and Figure 1B). This does not necessarily contradict with the hyperserotonemia previously reported in whole blood or platelet-rich plasma of patients with autism. The observed lower level can be explained on the basis that serotonin was measured in platelet-free plasma as the major source of blood serotonin. The obtained results are in good agreement with those of Spivak et al. [41] and Mostafa and Al-Ayadhi [42], who reported low levels of serotonin in platelet poor plasma and serum, respectively, in patients with autism compared to age-matched controls. Based on the fact that 99% of circulating serotonin is contained in platelets as a result of the function of the serotonin transporters [43], the hyperserotonemia previously reported in autistic whole blood or platelet-rich plasma and the recorded low serotonin in platelet-free plasma used in the present study, could be the result of i) increased serotonin production, or ii) increased expression of the transporter on platelet surface, or both. This is consistent with the previous work of Marazziti et al. [44], in which a significantly higher density of serotonin transporters per binding site was recorded in autistic compared to control subjects. In the present study, the lack of biochemical correlation between low serotonin levels in platelet-free plasma and severity of autism (SRS and CARS), could ascertain the role of platelet-related hyperserotonemia in the pathogenicity of this disorder [45]. This suggestion is in agreement with the previous work of Kuperman et al. [46], who recorded that although there was no significant correlation between platelet-rich serotonin concentration and autism behavior checklist scores, four individual items on the checklist that reflect behavioral abnormality were significantly associated with serotonin concentration. The relationship between serotonin levels and the behavioral symptoms in ASDs is of particular interest because it focuses on symptoms (e.g., repetitive and sensory circumscribed interests) that may respond to serotonergic medications.

Dopamine is a catecholamine synthesized from the dietary amino acid tyrosine. Once released from the neuron, central dopamine is broken down into homovanillic acid (HVA) and 3,4-dihydroxyphenylacetic acid. It is well documented that the dopaminergic system has been associated with speech and communication skills which are impaired in autism [47]. Approximately 50% of subjects with autism exhibited significantly elevated levels of HVA in CSF [48]. In the present study, Table 1 and Figure 1C demonstrate decreased plasma dopamine levels in patients with autism compared to control subjects. This is not consistent with previous studies in which assessment of circulating dopamine suggest possible elevations of whole-blood or plasma dopamine levels in children with ASD [49], with no consistent differences in the level of the dopamine metabolite HVA [50]. While increased levels have been associated with increased motoric and tic-like behavior, decreased dopamine levels have been linked to impaired communication and information processing [51]. The low level of plasma dopamine observed in the present study could be due to increased urinary excretion of HVA observed in children with autism [9]. Low plasma dopamine could be attributed to a remarkably higher rate of dopamine breakdown in Saudi individuals with autism compared to control subjects. The controversies between the present work and the previous work of El-Ansary et al. [12] on the same Saudi population could be solved and accepted through considering the pharmacologic effects of dopamine antagonistic drugs. While haloperidol and risperidone, which are used to treat aggressive symptoms in individuals with autistic aggressive symptoms and behaviors, were found to induce hyperactivity in patients with autism [52], administration of secretin (a peptide hormone) is associated with increases in CSFHVA levels and improvements in communication and reciprocal social interaction patterns [53]. This could help to conclude that alteration of dopamine homeostasis may confer risk of autism and this could, in turn, explain the absence of association between dopamine levels and the severity of SRS and CARS recorded in the present study (Table 1 and Figure 1C); in other words, either elevated or decreased levels are involved in the etiology of autism. This can be attributed to the differences in the distribution and abundance of dopamine receptors, reuptake transporters, and auto-receptors in the basal ganglia and prefrontal cortex involved in cognition function. A working hypothesis states that dopamine in these two brain areas regulates the balance between two functionally opponent processes (stability versus flexibility) and proposes that there is neurochemical reciprocity between dopamine in the prefrontal cortex and dopamine in the basal ganglia, with increases and decreases in prefrontal dopamine being associated, respectively, with decreases and increases in terms of dopamine in the basal ganglia [54,55]. This suggestion is consistent with a most recent study in which de novo missense mutation in the human dopamine transporter gene is identified as a risk factor for autism. The dopamine transporter is a presynaptic membrane protein that regulates dopaminergic tone in the CNS by mediating the high-affinity reuptake of synaptically released dopamine, making it a crucial regulator of dopamine homeostasis [55].

Another explanation which can be related to the role of dopamine in early brain development and etiology of autism is the conversion of dopamine into norepinephrine, which is catalyzed by dopamine β-hydroxylase [56] an enzyme controlled largely by a single gene, DβH. Robinson et al. [57] suggested that lower levels of DβH activity results in prenatal conditions that contribute to autism. Although they could not identify a mechanism, the authors raise the possibility that prenatal exposure to excessive dopamine levels could cause later down-regulation of dopamine production or dopamine sensitivity, with lasting effects throughout development. This could explain the reported low level of dopamine in the Saudi individuals with autism who participated in the present study.

Given that oxytocin is involved in the regulation of repetitive and affiliative behaviors, and that these are key features of autism, it is believed that oxytocin may play a role in ASD. Additionally, oxytocin is released into blood and within distinct brain regions in response to stressful and social stimuli, and has been shown to have an antidepressant-like effect in animal studies. Clinical reports suggest oxytocin to be a promising drug for psychiatric diseases such as depression, anxiety disorders, schizophrenia, and ASD. Oxytocin may also have therapeutic potential in the treatment of major depressive disorders, even though oxytocin administered into blood does not readily cross the blood-brain barrier [58].

Table 1 and Figure 1D show that autistic children have lower plasma oxytocin compared to age- and gender-matched control subjects. This is in agreement with several previous studies which demonstrate remarkable lower oxytocin levels in autistics compared to control subjects and suggest that oxytocin may play a role in the symptoms of ASDs [59-61]. Oxytocin has been implicated in the regulation of behavior in animals, but has not yet been examined in depth in autistic children. A profound impairment in social recognition in oxytocin receptor-knockout mice has been shown, indicating an important role played by these peptides in social and affective disorders, including autism and anxiety disorders [62]. The animals behaved normally, except they could not learn to recognize other mice or recognize their mother’s smell, though their sense of smell was normal. A single dose of oxytocin into the brain, however, cured the mice. This information could support the significant positive correlation recorded in the present study between lower levels of oxytocin in Saudi autistic subjects and the severity of the SRS score. This correlation is consistent with the previous work of Hoge et al. [63] and El-Masry et al. [64], which provide preliminary support for a link between social anxiety severity and plasma oxytocin. In a review by Lukas and Neumann [62], the regulatory capacity of oxytocin to modulate social behaviors in various rodent species implies a high translational potential. It also suggests that the brain oxytocin and arginine vasopressin systems are promising pharmaco therapeutic targets to improve social behavior and to reverse social deficits. Genetic evidence also supports the role of oxytocin in the pathophysiology of autism reported in the present study and the correlation presented between oxytocin and severity of autism. In a study by Yrigollen et al. [65], a link was found between the autism phenotype and both the oxytocin gene (OXT, located at 20p13), and the oxytocin receptor gene (OXTR, located at 3p26).

IFNs are a family of cytokines [66] which includes Type I (IFN-α and IFN-β) and Type II (IFN-γ) IFNs, among others [67,68]. Binding of an IFN (α, β, or γ) to the corresponding cell surface receptor results in activation of a family of tyrosine kinases and activation of signal transducer and activator of transcription (STAT) proteins [67]. Importantly, transcriptional activation of IFN stimulated genes by the activated STATs results in induction of IFN-inducible proteins such as IFI16. Recently, the role of IFI16 in recruiting inflammatory monocytes after Herpes simplex virus type 1 was ascertained [68].

Table 1 and Figure 1E demonstrate the significant increased levels of plasma IFI16 in children with autism compared to controls and significant correlation between high levels of IFI16 and severity of CARS and SRS. This means that concentrations of IFI16 in plasma of severe autistic subjects in CARS and SRS were remarkably higher than mild-moderate patients in both scores. This is consistent with the previous work of Garbett et al. [69], in which expression profiling of the superior temporal gyrus of six autistic subjects and matched controls revealed increased transcript levels of many immune system-related genes; IFI16 was one of the most significantly elevated transcripts. Overall, these expression patterns were found to be associated with the late recovery phase of autoimmune brain disorders. The remarkable elevation of IFI16 in Saudi individuals with autism as subjects of the present study could be supported through consideration of the recent work of El-Ansary and Al-Ayadhi [30], in which they recorded high significant increases of IFN-γ as inducer to IFI16. In addition, elevation of IFI16 in patients with autism compared to control subjects could also ascertain the role of autoimmunity in the pathophysiology of autism as, interestingly, anti-IFI16 antibodies are present in autoimmune diseases such as systemic lupus erythematosus. Hence, IFI16 may have a role not only in anti-viral responses, but also in pathogenic autoimmune responses [70].

Recently, the pro-apoptotic activity of IFI16 was reported by Gugliesi et al. [71]. They proved that IFI16 contributes in the regulation of apoptosis triggered by IFN Type I and that overexpression of IFI16 in primary endothelial cells is sufficient to induce apoptosis through the simultaneous activation of caspase 2 and caspase 3, which in turn is mediated by the NF-kB complex, since the inactivation of NF-kB was found to prevent IFI16-induced apoptosis. This information, together with the recorded elevation of IFI16 in patients with autism compared to control subjects, could be supported by the recent work of El-Ansary et al. [28] and El-Ansary Al-Ayadhi [30], in which they recorded elevation of caspase 3 and caspase 7 as pro-apoptotic markers in plasma of Saudi autistic children. The proposed relationship between NF-kB and the proinflammatory and proapoptotic effects of IFI16 could be supported by the work of Lam et al. [72], in which they suggested that obstetric complications in the form of perinatal hypoxia may lead to inflammation and apoptosis, and may contribute to an increased risk of neurodevelopmental disorders such as autism through increased expression of transcription factors, NF-κB, inflammatory mediators, IL-6, and the apoptotic protein caspase3. In contrast, this proposed relationship is not consistent with the recent finding of Malik et al. [73], which suggests that the NF-κB signaling pathway is not dysregulated in the brain of autistic subjects and thus may not be significantly involved in the processes of abnormal inflammatory responses suggested in the autistic brain.

Based on the fact that the predictiveness curve is better if it is farther away from the prevalence line and useless if it is close to the prevalence line, the predictiveness curves of the four measured parameters (Figure 2A–E), varies significantly from the baseline risk depending on whether GABA, serotonin, dopamine, oxytocin, and IFI16 concentrations were low or very high. This shows their usefulness as predictive biomarkers. This is supported by the high sensitivity and specificity recorded through ROC analysis (Table 2).

In conclusion, based on the obtained and discussed data, this study highlights the role of the investigated parameters representing inflammation, autoimmunity, and neurochemistry signaling pathways in the etiology and severity of ASD as an increasingly prevalent disorder. GABA, oxytocin, and IFI16 can be used as biochemical correlates to social and cognitive impairment in autism. In addition, the significant positive correlation between IFI16 and GABA together with the negative correlations between this inflammatory marker and serotonin, dopamine, and oxytocin (Figure 3) can support the role of inflammation in the neurochemical dysregulation recorded in patients with autism. Although initial reports look promising, the clinical application of the recorded biomarkers remains a vision for the future, since crucial questions have to be addressed first before these biomarkers find their way into clinical practice. Until the clinical use of the biomarkers become a reality, this study could help in the early detection of infants flagged at risk for autism and subsequently may likewise benefit from early behavioral intervention, making use of operant conditioning models, i.e., positive and negative reinforcement, to modify undesired behaviors.

## Abbreviations

ASDs: Autism spectrum disorders; CARS: Childhood autism rating scale; CNS: Central nervous system; CSF: Cerebrospinal fluid; GABA: Gamma aminobutyric acid; HRP: Horseradish peroxidase; HVA: Homovanillic acid; IFI16: Interferon-γ-induced protein-16; IFN: Interferon; ROC: Receiver operating characteristic; SRS: Social responsiveness scale; TNF-α: Tumor necrosis factor-α.

## Competing interests

The authors declare that they have no competing interests.

## Authors’ contributions

AA performed the biochemical assays; LA provided blood samples, and confirmed diagnosis and SRS and CARS scores; AE designed the work and drafted the manuscript. All authors read and approved the final manuscript.
